# Minimally invasive laser treatment combined with intravitreal injection of anti-vascular endothelial growth factor for diabetic macular oedema

**DOI:** 10.1038/s41598-019-44130-5

**Published:** 2019-05-20

**Authors:** Keiji Inagaki, Masafumi Hamada, Kishiko Ohkoshi

**Affiliations:** Department of Ophthalmology, St. Luke’s International Hospital, Tokyo, 104-8560 Japan

**Keywords:** Adaptive clinical trial, Retinal diseases

## Abstract

The purpose of this study was to investigate the effect of the combination of minimally invasive laser treatment to the intravitreal injection of anti-vascular endothelial growth factor (VEGF) for diabetic macular oedema (DME). This study was retrospective longitudinal study of thirty-four eyes of 31 patients with DME. Either once or several times of intravitreal anti-VEGF injection was followed by the single minimally invasive laser within a month. The mean best corrected visual acuity (VA) and the central macular thickness (CMT) were measured before treatment, 1, 3, 6 and 12 months after the first anti-VEGF injection. The mean logMAR VA had improved from 0.52 ± 0.34 at baseline to 0.44 ± 0.32 (p = 0.003), 0.40 ± 0.34 (p = 0.006), 0.43 ± 0.33 (p = 0.063), and 0.41 ± 0.34 (p = 0.009), at 1, 3, 6, and 12 months after treatment, respectively. The mean CMT decreased significantly by 1 month and maintained over 12 months (491.1 ± 133.9 µm at baseline, 396.6 ± 116.8 µm (p = 0.001), 385.2 ± 156.2 µm (p = 0.002), 336.5 ± 86.3 µm (p = 0.000), and 354.8 ± 120.4 µm (p = 0.000) at 1, 3, 6, and 12 months, respectively). The average number of the anti-VEGF injection in 1 year was 3.6 ± 2.1 in all patients. The combined intravitreal anti-VEGF and minimally invasive laser therapy improves the VA, alleviates DME, and may decrease the required number of anti-VEGF injections.

## Introduction

Laser photocoagulation had been the only evidence-based treatment for diabetic macular oedema (DME) since the Early Treatment Diabetic Retinopathy Study (ETDRS) report was published in 1985^1^. At present, however, anti-vascular endothelial growth factor (VEGF) agents constitute the first-line treatment for DME. The first anti-VEGF drugs were approved in 2014 in Japan, and they have proven to be effective in improving visual acuity (VA) and reducing macular oedema^2^.

One disadvantage of anti-VEGF monotherapy is the high frequency of injections per year. Patients require as many as eight injections a year, and this places a huge economic burden on patients and healthcare systems^2^. Recent studies have reported that the combination of anti-VEGF therapy with laser photocoagulation reduces the number of injections required^3^. On the basis of these promising findings, laser photocoagulation has again attracted attention as a treatment for DME.

However, some of the disadvantages of laser photocoagulation were listed as atrophic creep, emergence of scotoma due to heat-induced destruction of the retina, and restriction of effects to the maintenance of visual acuity^4–6^. To overcome these disadvantages, minimally invasive laser devices that leave no coagulation spots in the fundus have been developed over the past decade, with subthreshold micropulse laser photocoagulation and endpoint management using the PASCAL laser being introduced in recent years. These techniques reduce oedema without scarring in the macular area. Although the mechanism of conventional grid pattern photocoagulation has not been elucidated, destruction of the retinal pigment epithelium (RPE) and outer retinal layer presumably decreases oxygen consumption by the outer retina. This consequently results in an increase in the oxygen supply from the choroid to the inner retina^7^. Subthreshold photocoagulation is believed to irradiate the RPE and provide heat stimulation without destroying the tissue; thus, the term “photostimulation,” rather than “photocoagulation,” was proposed in a recent study^8^. We previously reported that subthreshold micropulse photocoagulation effectively maintained visual acuity and reduced macular oedema without damaging the outer retina in Japanese patients^9–14^.

Minimally invasive laser treatment has the large potential to reduce the burden of anti-VEGF injection under a combined use for the treatment of DME^15^. However, still very few studies have evaluated the outcomes of combination^15^. Therefore, we conducted a retrospective investigation of our patients with DME on the efficacy of the combination therapy.

## Materials and Methods

This was a retrospective study based on a review of medical records. The study protocol followed the tenets of the Declaration of Helsinki and was approved by the ethics committee of St. Luke’s International University. Informed consent for the analysis and use of treatment results was obtained from all patients at the time of the first visit.

In total, 34 eyes of 31 patients with DME—defined as clinically significant macular oedema according to the ETDRS criteria accompanied by diffuse and/or localized leakage from microaneurysms on fluorescein angiography— who have received intravitreal anti-VEGF injections and a minimally invasive laser therapy at St. Luke’s International Hospital between March in 2014 and September in 2015 were involved in this study. Eyes with the pre-treatment visual acuity ≤0.1 in logMAR (≥0.8 in decimal) or dense cataract, the eyes that had underwent intraocular surgeries, such as cataract surgery and vitreous surgery, or other treatments for macular oedema, such as intravitreal anti-VEGF injection or sub-Tenon’s capsule injection of triamcinolone, within the last 3 months were excluded. Among all eyes, 24 eyes of 22 patients with logMAR VA ≥ 0.3 (≤0.5 in decimal) were classified as a poor visual acuity group for a subgroup analysis.

### General treatment protocol

All eyes initially received intravitreal anti-VEGF injection either once or multiple times on a monthly basis, followed by the single minimally invasive laser irradiation within a month after the latest injection. Initial series of injection before laser treatment was conducted until the macular oedema is disappeared on OCT. The further anti-VEGF treatment was determined with a Pro re nata (PRN) regimen under monthly control over 12-month follow up period after the first injection.

Retreatment criteria were based on reduction in BCVA or an increase of CMT with approximately 20% or more, or presence of sub macular fluid. Patients who did not wish the loading phase due to economic problems or patients with mild macular oedema (less than 350 µm) did not completed loading phase.

### Minimally invasive laser therapy

The IQ577 (577 nm, OcuLight SLx, Iridex; Mountain View, CA, USA) and the PASCAL Streamline Yellow (xxx nm, Topcon Medical Laser Systems, Inc.; Santa Clara, CA, USA) were used as a minimally invasive micropulse laser therapy and a minimally invasive continuous wave laser endpoint management, respectively.

#### Minimally invasive micropulse laser therapy with IQ577

Before the treatment irradiations, test irradiations were performed at a non-oedematous area outside the vascular arcade to determine the threshold laser power for the retinal whitening, the power with which barely visible spots are observed approximately 3 s after irradiation. For that, the irradiation condition of the laser was chosen as follows; a spot diameter of 100 µm, total duration of 0.2 s, and 5% duty cycle. The irradiation power for treatment was set to 50% to 60% of the threshold value determined in test irradiations. The areas of oedema with a strong fluorescence leakage was irradiated with zero spacing. The used parameters for the titration were the power ranged from 850 to 1000 mW (t = 0.2 s, 5% duty cycle).

Direct micropulse coagulation for microaneurysms was performed using the IQ577, 15% duty cycle, 50-µm spot diameter, and 0.2-s duration. The leaking microaneurysms ≥500 µm away from the fovea defined with a fluorescein angiography was irradiated with a power which a hint of microaneurysm colour change was observed.

#### Minimally invasive continuous wave laser therapy with PASCAL Streamline Yellow (endpoint management)

As in the micropulse laser, test irradiations were performed for the energy titration at a non-oedematous area outside the vascular arcade. The minimum power at which coagulation spots were slightly visible approximately 3 s after irradiation with an irradiation diameter of 200 µm and duration of 0.015 s was set as the threshold. The threshold ranged from approximately 100 to 130 mW under these conditions. Then, the endpoint management was set to 30%–50% and irradiation was performed using a macular grid pattern (donut pattern) with the spacing of 0.5. The areas that could not be covered by the donut pattern were treated with additional coagulation using a 2 × 2 pattern.

When a non-perfusion area was observed outside the archade vessels on fluorescein angiography, we performed targeted retinal photocoagulation with a pattern scan laser using the PASCAL Streamline Yellow (Topcon Medical Systems, Inc.).

### Clinical examinations

The visual acuity (VA) and central macular thickness (CMT) were measured before treatment and 1, 3, 6, and 12 months after treatment. CMT was measured using optical coherence tomography (OCT). The decimal visual acuity was measured and converted to the logarithm of the minimum angle of resolution (logMAR) values for the analysis.

### Statistical analysis

For statistical analysis of changes in the VA and CMT after treatment, we used the Wilcoxon signed-rank test and the Friedman test. A p-value of <0.05 was considered statistically significant.

### Ethical approval

All procedures performed in studies involving human participants were in accordance with the ethical standards of the institutional and/or national research committee and with the 1964 Helsinki declaration and its later amendments or comparable ethical standards.

## Results

The mean age of the study subjects was 66.1 ± 6.0 years. The age range was 53 to 79 years old. There were 23 males (24 eyes) and eight females (10 eyes). All patients had type 2 diabetes, and the mean pre-treatment haemoglobin A1c value was 7.1% ± 1.2%. Nine eyes (26.5%, 9/34) belonged to patients with nephropathy, while two (5.9%, 2/34) belonged to patients undergoing dialysis. Retinal diseases included simple retinopathy (17.6%, 6/34), pre-proliferative retinopathy (32.4%, 11/34), and proliferative retinopathy (50%, 17/34). Treatment histories prior to the initiation of anti-VEGF therapy are shown in Table 1.

**Table 1:** Medical histories prior to anti-VEGF therapy.

Medical history	Number of eyes
Panretinal photocoagulation	23/34 (67.6%)
Conventional macular photocoagulation	1/34 (2.9%)
Subthreshold micropulse laser photocoagulation	6/34 (17.6%)
Subthreshold photocoagulation with Pascal Endpoint management	4/34 (11.8%)
Triamcinolone subtenon injection	3/34 (8.8%)
Intravitreal bevacizumab injection	7/34 (20.6%)
Vitreous surgery	1/34 (2.9%)
Cataract surgery	12/34 (35.3%)

Macular oedema included cystoid macular oedema alone (61.7%, 21/34), serous retinal detachment alone (8.8%, 3/34), complication of cystoid macular oedema and serous retinal detachment (26.5%, 9/34), and epiretinal membrane or vitreous traction (2.9%, 1/34). Parafoveal hard exudates were observed in nine eyes (26.5%, 9/34).

### Intravitreal anti-VEGF injections

Ranibizumab (79.4%, 27/34) or aflibercept (20.6%, 7/34) was used for the first injection. The number of the anti-VEGF injection prior to the laser irradiation was as follows; one (29.4%, 10/34), two (11.8%, 4/34), three (50%, 17/34), four (5.9%, 2/34), or six (1.8%, 1/34). Overall, the mean number of injections administered over 1 year was 3.6 ± 2.1. Ranibizumab treatment was switched to aflibercept treatment for five of the 34 eyes (14.7%). In the poor visual acuity group, the mean number of injections over 1 year was 4.0 ± 1.9. Ranibizumab treatment was switched to aflibercept treatment for five of the 24 eyes (20.8%).

### Minimally invasive laser treatment

The IQ577 and PASCAL Streamline Yellow were used for 19 (55.9%) and 15 (44.1%) eyes, respectively. The laser models and treatments are shown in Table 2.

**Table 2:** Laser devices used for the treatment of diabetic macular oedema.

Medical device	Number of eyes
**IQ577**	**19/34 (55**.**9%)**
Subthreshold grid pattern laser with Texcell mode	19/19 (100%)
Threshold coagulation for microaneurysms	11/19 (32.4%)
**PASCAL Streamline Yellow**	**15/34 (44**.**1%)**
Endpoint management (subthreshold pattern scanning laser)	13/15 (86.7%)
Pattern scan grid laser with threshold coagulation	2/15 (13.3%)
Targeted laser photocoagulation with pattern scanning laser	1/15 (6.7%)

### Post-treatment visual acuity changes

**Figure 1:**
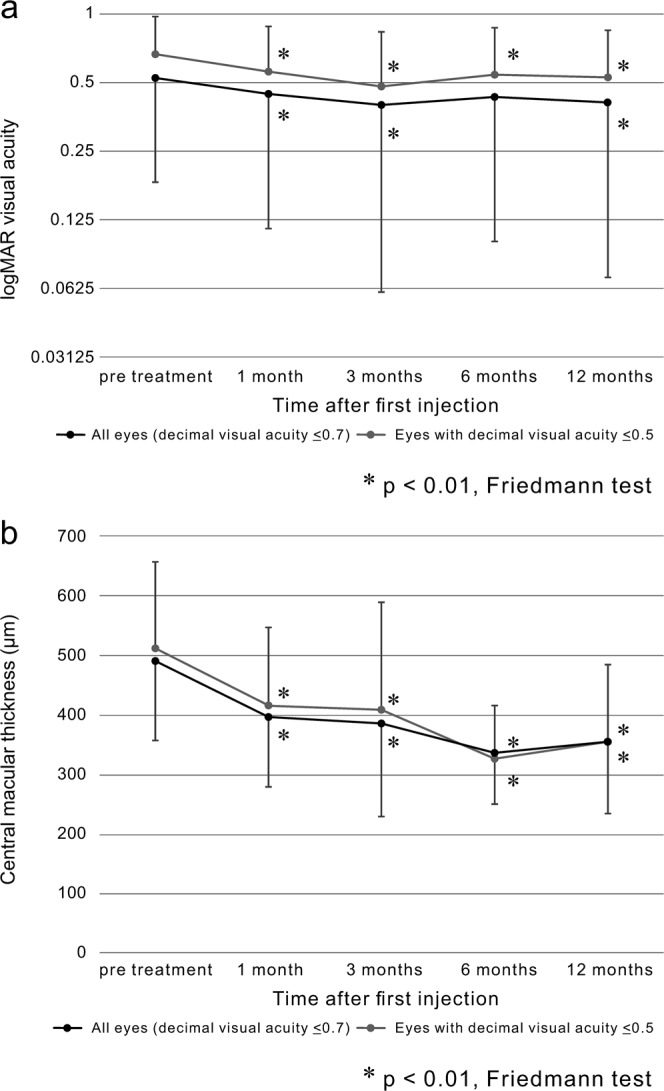
(**a**) Changes in the VA (logMAR) and (**b**) the CMT after combined laser and anti-VEGF therapy for patients with diabetic macular oedema: (**a**) VA was significantly improved at 1, 3, and 12 months after treatment (p = 0.003, p = 0.006, and p = 0.009, respectively). In addition, a significant improvement was observed in the subgroup with worse baseline VA (logMAR VA > 0.3) 1 month after treatment, which was maintained 12 months after treatment. (**b**) CMT was considerably reduced 1 month after treatment, and the improvement was maintained 12 months after treatment (p < 0.001). Eyes with decimal visual acuities ≤0.5 also showed significant decreases in oedema 1 month after treatment, with maintenance of this improvement 12 months after treatment (p < 0.05).

Overall, the mean logMAR VA was 0.52 ± 0.34 at baseline, 0.44 ± 0.33, 0.40 ± 0.34, 0.43 ± 0.33, and 0.41 ± 0.34 at 1, 3, 6, and 12 months after treatment, respectively (p = 0.003, 0.006, 0.063, and 0.009, respectively). The VA of eyes of the subgroup with the poor baseline-VA also showed a significant improvement by 1 month after treatment, which was maintained 12 months after the treatment (0.66 ± 0.31 at baseline, 0.56 ± 0.33, 0.48 ± 0.36, 0.54 ± 0.32, and 0.54 ± 0.33, with a p-value of 0.001, 0.001, 0.017, and 0.025, at 1, 3, 6, 12 months after treatment, respectively) (Fig. 1a).

An increase of ≥0.3 logMAR at 12 months after the treatment was observed for seven of the 34 eyes (20.6%). The VA remained unchanged for 25 (73.5%) and deteriorated for two (5.9%) of the 34 eyes. Of the 24 eyes with poor baseline-VA, seven (29.1%) showed an increase of ≥0.3 logMAR, 15 (62.5%) showed no change, and two (8.3%) showed deterioration.

### Post-treatment changes in macular oedema

The overall mean CMT was 491.1 ± 133.9 µm before treatment, whereas it was 396.6 ± 116.8 µm (p = 0.001), 385.2 ± 156.2 µm (p = 0.002), 336.5 ± 86.3 µm (p = 0.000), and 354.8 ± 120.4 µm (p = 0.000) at 1, 3, 6, and 12 months after treatment, respectively. A significant reduction in the CMT was observed at 1 month, and this improvement was maintained over 12 months. The subgroup of the worse baseline VA also exhibited a significant reduction in oedema at 1 month, which was maintained at 12 months (511.2 ± 143.9 µm, 416.7 ± 129.9 µm (p = 0.001), 408.3 ± 181.1 µm (p = 0.010), 325.9 ± 89.0 µm (p = 0.000), 354.5 ± 130.4 µm (p = 0.000), baseline, after 1, 3, 6, and 12 months, respectively) (Fig. 1b).

Three months after treatment, CMT was reduced by ≥20% in 18 of 32 eyes (56.2%), while it remained unchanged in 10 eyes (31.3%) and had increased in four eyes (12.5%). Twelve months after treatment, a reduction of ≥20% was observed in 20 of 33 eyes (60.6%), with no change in 12 eyes (36.4%), and an increase in one eye (3.0%).

### Improvement in the mean VA and the mean number of intravitreal anti-VEGF injections in the one-year follow-up period

Improvements in the mean VA and the mean number of intravitreal anti-VEGF injections in 1-year follow-up period in the entire study cohort and in the poor visual acuity subgroup are shown in Table 3. The mean VA had improved by 0.12 logMAR (corresponding to 5.9 ETDRS letters) in the entire cohort and by 0.14 logMAR (corresponding to 6.9 ETDRS letters) in the poor visual acuity subgroup. The mean numbers of anti-VEGF injections were 3.6 and 4.0, respectively.

**Table 3:** Improvement in the mean visual acuity and the mean number of antivascular endothelial growth factor (anti-VEGF) injections 1 year after combined anti-VEGF and laser therapy for patients with diabetic macular oedema.

	Entire study cohort	Patients with initial decimal visual acuities ≤0.5
LogMAR	0.12	0.14
ETDRS	5.9 letters	6.9 letters
Number of injections	3.6	4

logMAR: logarithm of the minimum angle of resolution, ETDRS: Early Treatment of Diabetic Retinopathy Study.

### Comparison of the mean VA, CMT, and number of intravitreal anti-VEGF injections between the IQ577 group and PASCAL group

Comparison of the treatment results between the IQ577 group and PASCAL group is shown in Table 4. There was no difference between the groups in mean VA, CMT, and number of anti-VEGF injections in a year.

**Table 4:** Comparison of visual acuity, central macular thickness and number of injections in 1 year in IQ577 group vs. PASCAL group.

		IQ577 (subthreshold micropulse) group (mean ± SD) n = 19	PASCAL (minimally invasive continuous wave) group (mean ± SD) n = 15	p-value
LogMAR visual acuity	pre treatment	0.51 ± 0.33	0.55 ± 0.37	n.s
	1 month	0.4 ± 0.29	0.49 ± 0.37	n.s
	3 month	0.34 ± 0.28	0.46 ± 0.40	n.s
	6month	0.38 ± 0.31	0.50 ± 0.35	n.s
	12month	0.37 ± 0.32	0.46 ± 0.36	n.s
central macular thickness (µm)	pre treatment	476.58 ± 125.63	509.53 ± 145.92	n.s
	1 month	410.21 ± 134.63	378.14 ± 88.55	n.s
	3 month	383.47 ± 189.44	387.2 ± 113.96	n.s
	6month	326.47 ± 78.20	351.15 ± 98.46	n.s
	12month	346.05 ± 133.70	366.57 ± 103.24	n.s
number of injections in 1 year		3.95 ± 2.53	3.06 ± 1.39	n.s

logMAR: logarithm of minimum angle of resolution.

### Adverse effects/complications

Cerebral infarction was observed in one case (2.9%, 1/34) that received ranibizumab in the first injection. There were no ocular complications associated with laser treatment or intravitreal anti-VEGF injection.

### A representative case

**Figure 2:**
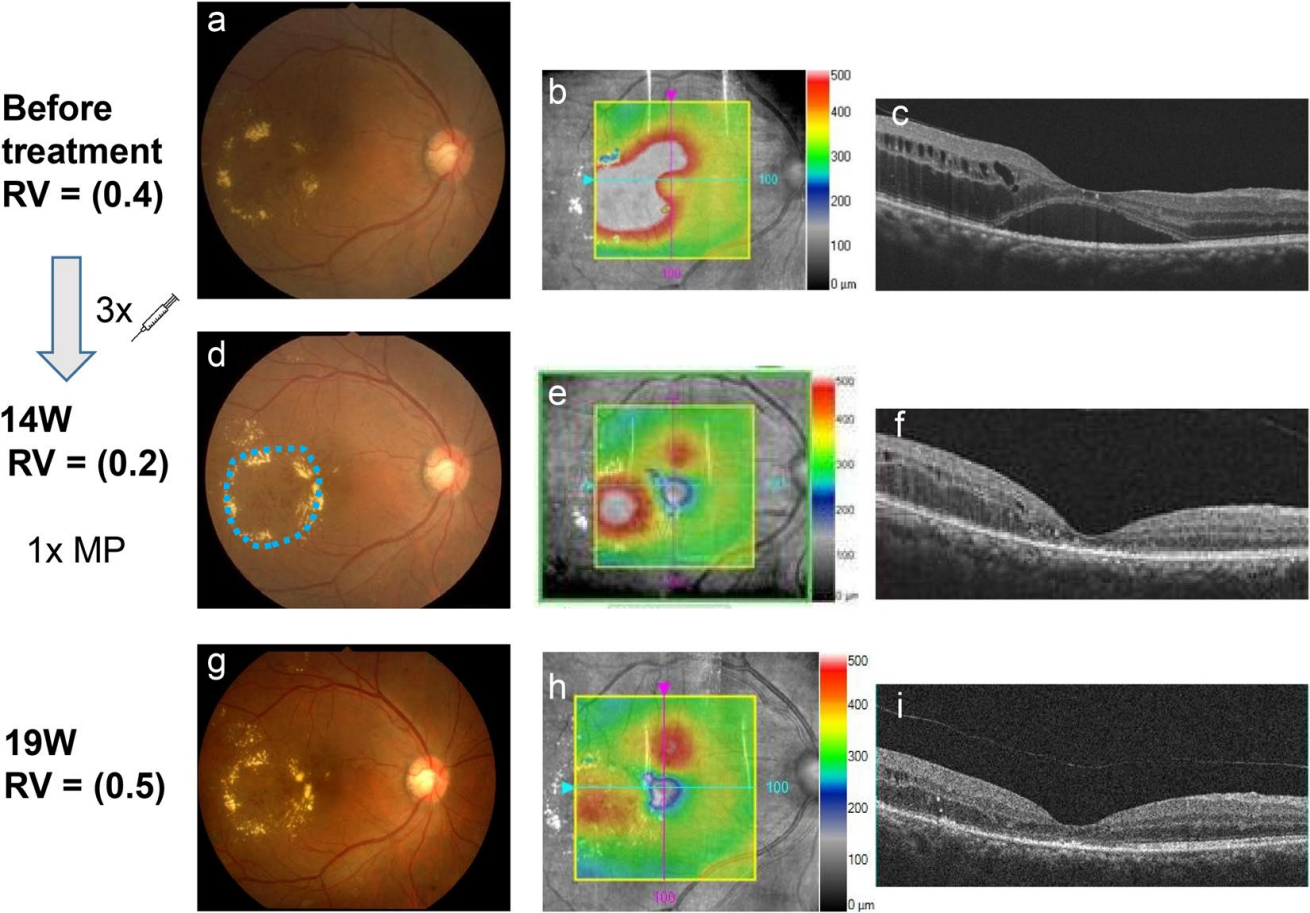
Representative case of a 58-year-old man with diabetic macular oedema treated with combined laser and anti-vascular endothelial growth factor (anti-VEGF) therapy. The patient exhibited diabetic macular oedema with serous retinal detachment in the fovea. His corrected visual acuity was 0.4 (**a**–**c**). Following the resolution of oedema after three intravitreal anti-VEGF injections, subthreshold micropulse laser coagulation and direct coagulation were performed to treat the residual temporal oedema (blue circle) (**d**) at week 14 (**d–f**). Nineteen weeks after the first injection, after three anti-VEGF injections and a single session of laser treatment, the oedema had disappeared, and the corrected visual acuity had improved to 0.5 (**g–i**). RV: Right vision.

A representative case is presented in Fig. 2. The patient was a 58-year-old man with focal serous retinal detachment in the fovea and circinate exudation with numerous microaneurysms on the temporal side of the fovea. The pre-treatment corrected decimal visual acuity was 0.4 (Fig. 2a–c). Following the reduction in oedema after three anti-VEGF injections, subthreshold micropulse laser coagulation using the IQ577 and direct coagulation were performed for the residual temporal oedema at week 14 (Fig. 2d–f). After laser application, macular oedema improved, and the corrected visual acuity had improved to 0.5 (Fig. 2g–i).

## Discussion

In this study, we evaluated the efficacy of intravitreal anti-VEGF injection combined with minimally invasive laser treatment for the treatment of DME and assessed the 12-month outcomes. The results showed that macular oedema was significantly reduced while visual acuity was significantly improved at 1 month after treatment, and that the improvements were maintained at 12 months after treatment.

Twelve months after treatment, the mean number of anti-VEGF injections and the corresponding ETDRS letter gain were 3.6 and 5.9, respectively, in the entire cohort, and 4 and 6.9, respectively, in the poor visual acuity subgroup. The corresponding values for the anti-VEGF monotherapy in previous studies were as follows: 10.2 and 10.3, respectively, in the RESOLVE study^16^, 7.8 and 6.6, respectively, in the REVEAL study^17^, and 7.4 and 7.9, respectively, in the RESTORE study^18^. Thus, the visual gain in the current study within 12 months after minimally invasive laser with intravitreal anti-VEGF injection was slightly worse than the average gain in previous prospective studies with anti-VEGF treatment alone. However, focusing on the number of injections, very small number of anti-VEGF injections (3.6) were required in our combination study group. The number of anti-VEGF injections required for treatment is often a burden for patients, both socially and economically. Therefore, one of the advantages of combined anti-VEGF and deferred laser therapy may lie in its potential to decrease the required number of anti-VEGF injections. A study with longer follow-up time is necessary to evaluate the effect of combination therapy on long-term visual outcome.

For several decades, laser monotherapy was the standard treatment strategy for DME. However, this treatment modality is associated with the side effect of deterioration of macular function. Subthreshold micropulse laser photocoagulation was introduced to overcome the side effects of focal macular laser therapy, and its efficacy for reducing macular oedema in DME is reported to be equal to or higher than that of conventional modified-ETDRS coagulation^19^. Furthermore, macular sensitivity is better with subthreshold micropulse photocoagulation than with conventional laser therapy^10^. Anti-VEGF therapy recently gained approval for DME treatment and is currently the standard treatment for this condition. Therefore, the indication of laser monotherapy is limited to focal macular oedema, which can be treated with subthreshold laser therapy alone. However, in cases of severe diffuse oedema, laser monotherapy is not recommended, considering known evidence regarding the efficacy of anti-VEGF therapy. Therefore, we decided to combine anti-VEGF and minimally invasive laser therapy for the treatment of patients with DME and investigated the efficacy of this combination treatment in the present study.

The literature shows that the combination of focal macular laser therapy and anti-VEGF injections, particularly for initial treatment, does not improve the visual acuity. The Diabetic Retinopathy Clinical Research Network (DRCR-net) documented an ETDRS letter gain of 7.2 when treatment was initiated as combination treatment (immediate laser group) and 9.8 letters when laser treatment was added after 6 months of anti-VEGF treatment (deferred laser group). Thus, the number of letters gained was larger in the deferred laser group^20^.

In contrast, Liegl *et al*.^3^ compared pro re nata (PRN) administration after three loading doses of anti-VEGF (anti-VEGF monotherapy) with NAVILAS laser therapy after three loading doses of anti-VEGF (anti-VEGF + laser combination) and showed that 84% eyes in the monotherapy group and 36% eyes in the combination therapy group required additional injections within 12 months. Furthermore, the improvement in visual acuity and reduction in CMT were not significantly different when comparing the anti-VEGF monotherapy group to the anti-VEGF + laser combination group. In the present study, we found that anti-VEGF + laser combination therapy had the potential to reduce the required number of intravitreal anti-VEGF injections.

The discrepancies in the results of the two abovementioned studies may be related to the timing of the laser therapy. It may be possible that laser therapy under a thickened retina induces severe thermal damage, and more laser energy is required to treat severe macular oedema, when the faint whitening of the retinal colour is used as the indicator of the effective burn in the conventional laser therapy. We posit that tissue damage resulting from laser therapy may account for a decreased visual acuity. In contrast, deferred laser therapy administered after a reduction in macular oedema results in the use of lower laser energy levels, potentially leading to visual improvements.

Intravitreal anti-VEGF drugs exert their effects on macular oedema soon after administration, following which they are cleared from the system. On the other hand, minimally invasive laser treatment, including subthreshold micropulse photocoagulation, has been shown to exert its effects 3 months after irradiation^9,11,13,14^. Thus, combination therapy allows for a persistent reduction in macular oedema after the effects of the anti-VEGF drug have attenuated because of clearance.

In the present study, laser treatment was intentionally applied after three loading doses of intravitreal anti-VEGF injections in 50% of patients. In addition to the advantages of reduced laser energy and persistent reduction in macular oedema, this treatment strategy has another technical advantage. In subthreshold laser treatment, the laser spot is invisible. Therefore, laser energy has to be determined using titration methods. Because titration is performed in the retina, where no oedema is present, there is always a discrepancy in the energy used at the laser site if macular oedema is present. If the laser application site is not oedematous, the energy is presumably set at the appropriate level.

Although the mechanism of subthreshold micropulse photocoagulation for macular oedema has not been elucidated, this treatment is considered to reduce macular oedema by providing heat stimulation without destroying the RPE. Thus, its mechanism of action is different from that of anti-VEGF therapy. In addition, unlike conventional laser treatment that destroys the structure of the retinal layers, this treatment modality is non-invasive and allows for additional irradiation^8,13^. The endpoint management irradiation system with the PASCAL laser automatically calculates coagulation conditions via a unique algorithm for performing grid pattern scan laser photocoagulation, which enables simple and appropriate performance of subthreshold photocoagulation. This system is considered a minimally invasive laser treatment system that does not destroy the RPE, similar to subthreshold micropulse laser photocoagulation^21,22^.

Finally, no treatment-related complications, including the enlargement of coagulation spots and emergence of scotoma after laser irradiation, occurred in the present study. This suggests that the treatment efficacy and safety are better with a combination of minimally invasive laser therapy and intravitreal anti-VEGF injections than with a combination of conventional laser therapy and intravitreal anti-VEGF injection or a with a conventional laser therapy alone.

## Conclusions

The results of this study suggest that the combination of intravitreal anti-VEGF injections with minimally invasive laser therapy for DME effectively improves the visual acuity and reduces macular oedema by 1 year after treatment. In addition, it reduces the required number of intravitreal anti-VEGF injections, thus reducing the economic burden on patients and healthcare systems. It would be important to create an algorithm for the combination therapy. On the basis of this study, we recommend initial anti-VEGF therapy with a loading dose (3 or more) until macular oedema is almost resolved, followed by application of a minimally invasive laser for residual oedema. If macular oedema recurs, the initial dose (which may not require 3 loading doses) should be repeated.

However, considering the limitations of the small sample size, short follow-up period (12 months), and retrospective nonrandomized design, a long-term follow-up study with a larger sample size is essential to confirm our findings.

## Data Availability

The data used to support the findings of this study are included within the article.

## References

[1] Early Treatment of Diabetic Retinopathy Study Research Group. Photocoagulation for diabetic macular edema. Early treatment diabetic retinopathy study report number 1. *Arch*. *Ophthalmol*. **103**, 1796–1806 10.1001/archopht.1985.01050120030015 (1985). 2866759

[2] Diabetic Retinopathy Clinical Research Network. Aflibercept, bevacizumab, or ranibizumab for diabetic macular edema. *N*. *Engl*. *J*. *Med*. **372**, 1193–1203 10.1056/NEJMoa1414264 (2015). 10.1056/NEJMoa1414264 PMC4422053 25692915

[3] Liegl R (2014). Comparative evaluation of combined navigated laser photocoagulation and intravitreal ranibizumab in the treatment of diabetic macular edema. PLoS One..

[4] Schatz H, Madeira D, McDonald R, Johnson RN (1991). Progressive enlargement of laser scars following grid laser photocoagulation for diffuse diabetic macular edema. Arch. Ophthalmol..

[5] Guyer DR, D’Amico DJ, Smith CW (1992). Subretinal fibrosis after laser photocoagulation for diabetic macular edema. Am. J. Ophthalmol..

[6] Rutledge BK, Wallow IHL, Poulsen GL (1993). Sub-pigment epithelial membranes after photocoagulation for diabetic macular edema. Arch. Ophthalmol..

[7] Vujosevic S (2013). Subthreshold laser therapy for diabetic macular edema: metabolic and safety issues. Curr. Med. Chem..

[8] Inagaki Keiji, Shuo Takuya, Katakura Kanae, Ebihara Nobuyuki, Murakami Akira, Ohkoshi Kishiko (2015). Sublethal Photothermal Stimulation with a Micropulse Laser Induces Heat Shock Protein Expression in ARPE-19 Cells. Journal of Ophthalmology.

[9] Ohkoshi K, Yamaguchi T (2010). Subthreshold micropulse diode laser photocoagulation for diabetic macular edema in Japanese patients. Am. J. Ophthalmol.

[10] Hoshikawa Y, Ohkoshi K, Yamaguchi T (2011). A study of short-term changes in retinal sensitivity after subthreshold micropulse photocoagulation for diabetic macular edema. Nippon Ganka Gakkai Zasshi.

[11] Inagaki K, Ohkoshi K, Ohde S (2012). Spectral-domain optical coherence tomography imaging of retinal changes after conventional multicolor laser, subthreshold micropulse diode laser, or scanning laser therapy in Japanese with macular edema. Retina.

[12] Inagaki K (2015). Comparative efficacy of pure yellow (577-nm) and 810-nm subthreshold micropulse laser photocoagulation combined with yellow (561–577-nm) direct photocoagulation for diabetic macular edema. Jpn. J. Ophthalmol..

[13] Inagaki Keiji, Ohkoshi Kishiko, Ohde Sachiko, Deshpande Gautam A., Ebihara Nobuyuki, Murakami Akira (2014). Subthreshold Micropulse Photocoagulation for Persistent Macular Edema Secondary to Branch Retinal Vein Occlusion including Best-Corrected Visual Acuity Greater Than 20/40. Journal of Ophthalmology.

[14] Inagaki K, Iseda A, Ohkoshi K (2012). A study of outcomes of subthreshold micropulse diode laser photocoagulation combined with direct photocoagulation for diabetic macular edema. Nippon Ganka Gakkai Zasshi.

[15] Moisseiev E (2018). Subthreshold micropulse laser reduces anti-VEGF injection burden in patients with diabetic macular edema. Eur. J. Ophthalmol..

[16] Massin P (2010). Safety and efficacy of ranibizumab in diabetic macular edema (RESOLVE Study): a 12-month, randomized, controlled, double-masked, multicenter phase II study. Diabetes Care.

[17] Ishibashi T (2015). The REVEAL study: ranibizumab monotherapy or combined with laser versus laser monotherapy in Asian patients with diabetic macular edema. Ophthalmology.

[18] Schmidt-Erfurth U (2015). Three-year outcomes of individualized ranibizumab treatment in patients with diabetic macular edema: the RESTORE extension study. Ophthalmology.

[19] Lavinsky D (2011). Randomized clinical trial evaluating mETDRS versus normal or high-density micropulse photocoagulation for diabetic macular edema. Invest. Ophthalmol. Vis. Sci..

[20] Elman MJ (2015). Intravitreal ranibizumab for diabetic macular edema with prompt versus deferred laser treatment: 5-year randomized trial results. Ophthalmology.

[21] Sramek C (2011). Non-damaging retinal phototherapy: dynamic range of heat shock protein expression. Invest. Ophthalmol. Vis. Sci..

[22] Lavinsky D (2014). Subvisible retinal laser therapy: titration algorithm and tissue response. Retina.

